# Integrin α6/Akt/Erk signaling is essential for human breast cancer resistance to radiotherapy

**DOI:** 10.1038/srep33376

**Published:** 2016-09-14

**Authors:** Ting Hu, Rui Zhou, Yanxia Zhao, Gang Wu

**Affiliations:** 1 Cancer Center, Union Hospital, Tongji Medical College, Huazhong University of Science and Technology, 1277 JieFang Avenue, Wuhan 430022, China

## Abstract

Integrin α6 (ITGA6), a transmembrane glycoprotein adhesion receptor protein, is widely upregulated in many types of tumors and promotes migration and invasion in cancer cells. However, the role that the ITGA6-associated signaling network plays in radiosensitivity in breast cancer has not been described. The expression of ITGA6 was examined in human breast cancer and normal breast cell lines using western blot analysis. We also explored the role of ITGA6 in the regulation of radiation sensitivity in breast cancer using the colony formation assays, cell cycle analyses, apoptosis assays and immunofluorescence analyses. The results showed that the protein and mRNA expression levels of ITGA6 was higher in breast cancer cells than in normal cells. ITGA6 protectived responses to radiotherapy in breast cancer cells by altering cell apoptosis, DNA damage repair and cell-cycle regulation. Furthermore, ITGA6 enhanced radiation resistance via PI3K/Akt and MEK/Erk signaling. In addition, overexpressing ITGA6 promoted radiation resistance in cells, and this effect was neutralized by the PI3K inhibitor LY294002 and MEK inhibitor U0126. Taken together, these findings indicate that ITGA6 might be involved in a mechanism that underlies radiation resistance and that ITGA6 could be a potential target for therapies aimed at overcoming radiation resistance in breast cancer.

Malignant breast cancer is one of the most commonly diagnosed cancers worldwide and the second leading cause of cancer-related deaths among females^1^. Adjuvant radiotherapy reduces the risk of loco-regional relapse and provides a survival benefit in most patients following breast conserving surgery and in patients who are at high risk of recurrence following mastectomy^2^. However, a percentage of patients suffer loco-regional relapse after radiotherapy, and the failure to maintain local control of breast cancer decreases overall survival rate in these patients^3^. Radioresistance is the primary reason for failed treatment, and identifying a molecular signature that can predict the outcome of radiotherapy and factors that can be targeted to sensitize radioresistant cells are therefore essential for improving the efficacy of radiotherapy in breast cancer.

Integrin α6 (ITGA6), a transmembrane glycoprotein adhesion receptor that mediates cell-matrix and cell-cell adhesion^4^,^5^, is overexpressed in breast cancer tissue and cell lines and is associated with a poor prognosis and reduced survival rates^6^. In particular, when used alone, ITGA6 expression has been shown to be a better predictor of reduced survival than other known factors, including estrogen receptor status. These data indicate that inhibiting ITGA6 might be an effective strategy for improving survival. In addition, ITGA6 is a marker of cancer stem cells^7^,^8^,^9^. Several studies have shown that ITGA6 contributes to the regulation of processes including cell adhesion, migration, invasion and survival^10^,^11^,^12^,^13^. Studies targeting ITGA6 have demonstrated its strong potential to sensitize cancer cells to conventional radiotherapies in prostate and esophageal cancers^14^. However, no study has determined the relationship between ITGA6 and radiation sensitivity in breast cancer cells, and the molecular mechanism underlying how ITGA6 confers radioresistance to tumor cells remains unclear.

Integrins have been shown to regulate multiple intracellular signaling pathways, including the PI3K/Akt and MAPK/Erk pathways, by coupling with cytoplasmic kinases, small GTPases, and scaffolding proteins and by interacting with and modulating the activity of other receptors at the cell surface^15^. PI3K inhibitors have long been known to sensitize or to work in combination with IR to enhance apoptosis in breast cancer cells^16^. In addition, Akt is emerging as a central mediator of resistance^17^. Moreover, extensive evidence indicates that the activation of MAPK/Erk pathways is correlated with tumor progression, resistance to treatment and worse survival inpatients^18^,^19^.

In this study, three breast cancer cell lines with different molecular subtypes were used: MCF-7(HR+/HER2−), SKBR-3(HR−/HER2+) and MDA-MB-231(HR−/HER2−). We investigated the association between ITGA6 expression and susceptibility to radiotherapy. Our data shows that overexpressing ITGA6 induced resistance to radiotherapy and that this radio-resistant effect was mediated, at least in part, through the PI3K/Akt or MEK/Erk signaling pathways. Overexpression of ITGA6 affect processes including cellular anti-apoptosis, cell cycle arrest and DNA double-strand break repair in breast cancer cells. These data suggest that individualized care could be implemented based on phenotypic markers such as ITGA6 that reflect individual tumor characteristics. This type of strategy might therefore eventually be used to direct adjuvant treatment decisions by taking into account the intrinsic risks of locoregional relapse. Targeting ITGA6 signaling might be an effective strategy for using adjuvant radiotherapy in breast cancer.

## Results

### ITGA6 expression is increased in breast cancer

**Figure 1:**
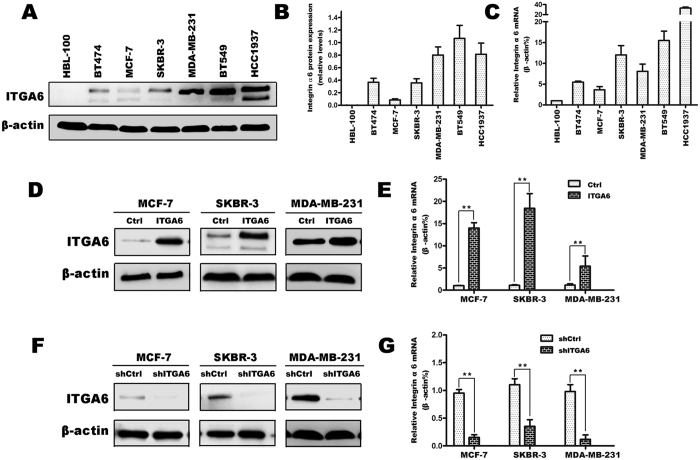
Protein and mRNA expression level of ITGA6 in breast cancer cell lines. (**A**,**B**) Western blot analysis of ITGA6 expression in HBL-100, MCF–7, MDA-MB-231, HCC1937, SKBR-3, BT474 and BT549 group with ITGA6 antibody. β-actin served as loading control. The intensity of ITGA6 was quantified by densitometry (software: Image J, NIH). (**C**) Relative ITGA6 mRNA expression levels were determined using RT-PCR. β-actin mRNA was used as the internal control. (**D**,**E**) Western blot and RT-PCR analysis to measure the protein and mRNA levels of ITGA6 in breast cancer cells transfected with LZRS-IRES-zeo-α6 and LZRS-IRES-zeo vectors for MCF7, SKBR-3 and MDA-MB-231 cells lines. (**F**,**G**) Western blot and RT-PCR analysis of ITGA6 in the ITGA6 knockdown and control shRNA group. All experiments were carried out in triplicate. Data are presented as mean ± SD (n = 3). **P < 0.01 in comparison with respective group.

To determine whether ITGA6 expression is associated with radiation sensitivity in breast cancer, we measured the expression of ITGA6 in six breast cancer cell lines, including MCF7 (HR+/HER2−), BT474 (HR+/HER2+), SKBR-3 (HR−/HER2+) and MDA-MB-231, BT549 and HCC1937 (HR−/HER2−) and determined their sensitivities to the radiotherapy. Our results showed that all the breast cell lines express increased levels of ITGA6 at both the mRNA and protein level, compared with normal mammary epithelial HBL-100 cells which only showed minimal ITGA6 expression levels. The highest levels were observed in the MDA-MB231, BT549 and HCC1937 cells, whereas relatively lower levels were observed in MCF7, BT474 and SKBR-3 cells (Fig. 1A–C). Expression was not significantly associated with HER2 status, but there was an apparently inverse relationship between ER expression and ITGA6 expression. The clonogenic formation assay showed that the percentage of cells that survived in MCF7 and SKBR-3 cancer cells was much higher than the HCC1937 and BT549 cancer cells. These results seem to indicate that ITGA6 expression is not correlated with survival when cells are exposed to radiation. Short hairpin RNA (shRNA) was used to knockdown ITGA6 expression in MCF7, SKBR-3 and MDA-MB-231 cells. Forty-eight hours after transfection, ITGA6 mRNA and protein were drastically decreased (Fig. 1F,G). In addition, LZRS-IRES-zeo control or LZRS-IRES-zeo-α6 retroviral vector were transiently transfected into both cell lines. After concentration of zeocin used to select stable cell lines, ITGA6 mRNA and protein was significantly overexpressed (Fig. 1D,E).

### ITGA6 expression was associated with radiation resistance *in vitro*

**Figure 2:**
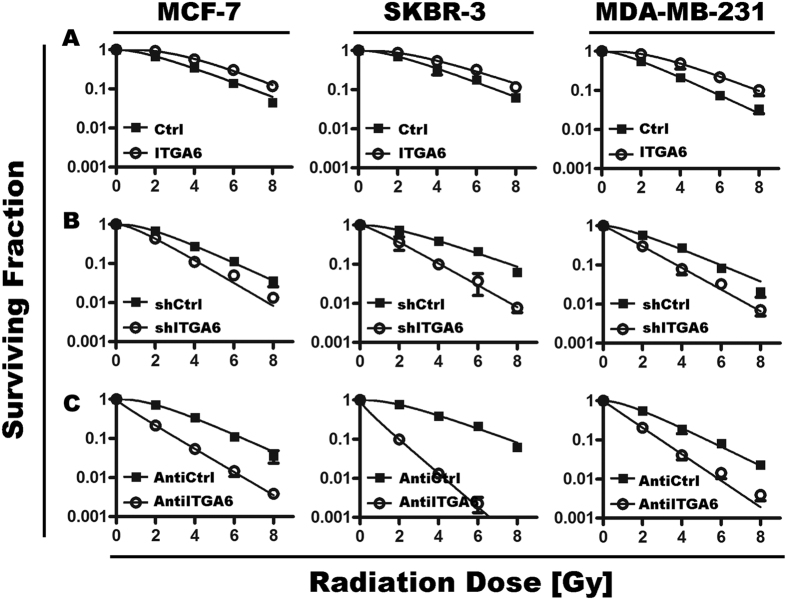
Effect of ITGA6 on radiation sensitivity of breast cancer cells. Clonogenic survival assays were performed as described in the Materials and Methods section. The y axis represents percentage colony formation relative to unirradiated cells of the respective groups; the x axis represents the various radiation doses. Each data point represents the mean ± SD of three independent experiments.

To further validate whether ITGA6 affects radiation sensitivity, cell viability was determined by Clonogenic Assay. Overexpression of ITGA6 increased clonogenic formation in MCF7, SKBR-3 and MDA-MB-231 cells compared with the cells transfected with the negative control (Fig. 2A). At the same time, we used 2 approaches to inhibit functional of ITGA6. First, we incubated cells with a blocking monoclonal antibody against CD49f (clone GoH3) to inhibit ITGA6 function. Alternatively, we knocked down the ITGA6 gene with shRNA technology. Both approaches induced the significant decline in clonogenic survival in irradiated cells at all applied radiation dose (Fig. 2B,C).

### ITGA6 interferes with irradiation-induced cell apoptosis

**Figure 3:**
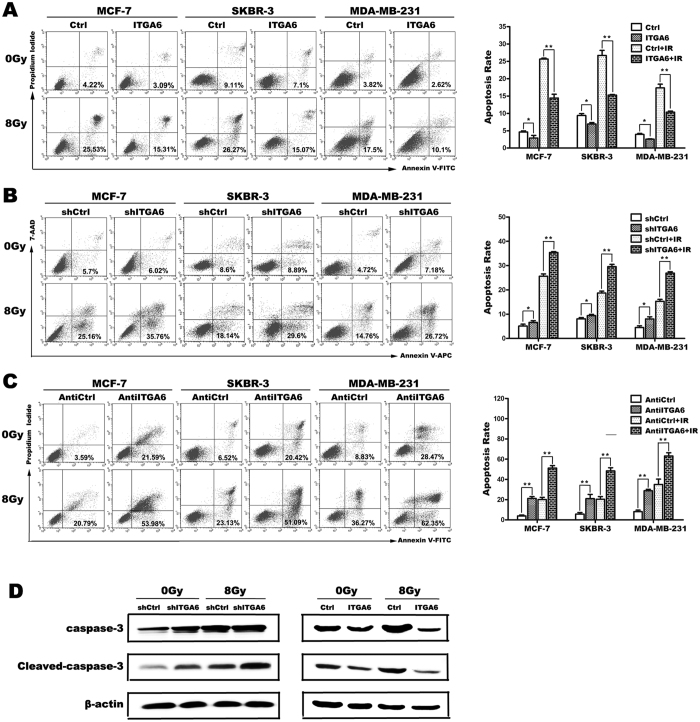
ITGA6 interferes with irradiation-induced cell apoptosis. (**A**–**C**) Images showing the flow cytometric analysis of apoptosis. Representative graphs are shown (early apoptosis, lower right area; and late apoptosis, upper right area). Histogram represents the percentage of cells exhibiting multiple nuclei following the treatments indicated as described in (**A**) **p < 0.01. The results are representative of three independent experiments. (**D**) The expression level of the caspase-3 and cleaved-caspase-3 protein was determined in the indicated cells using western blot analysis.

To evaluate whether the radioresistance resulting from ITGA6 knockdown was due to inhibition of radiation-induced apoptosis, we subsequently performed flow cytometry analysis to determine the role of ITGA6 in radiation-induced apoptosis. As expected, radiation-induced apoptosis is suppressed after inhibition of ITGA6 through both 2 approaches (Fig. 3B,C) in MCF7, SKBR-3 and MDA-MB-231 cells, whereas overexpression of ITGA6 upregulates cell apoptosis (Fig. 3A).

To confirm these findings, we detected the apoptotic effector caspase-3 and cleaved caspase-3 protein. The results showed that both caspase-3 and cleaved caspase-3 protein levels were markedly up-regulated after radiation treatment in the control cells. Whereas, caspase-3 and cleaved caspase-3 protein were much lower in the cells that stably overexpressed ITGA6 (Fig. 3D). Inhibiting ITGA6 therefore increased caspase-3 and cleaved caspase-3 expression in response to irradiation. These results indicate that ITGA6-mediated resistance to radiation involves the inhibition of radio-induced apoptosis.

### ITGA6 depletion prolonged IR-induced G2/M arrest

**Figure 4:**
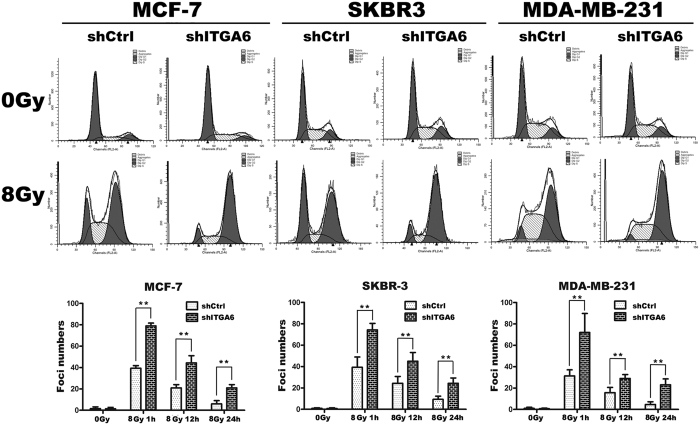
ITGA6 depletion prolonged IR-induced G2/M arrest. Following the protocol described in method, MCF7, SKBR-3 and MDA-MB-231 cells were transfected with shRNA as indicated and left untreated or treated with 8 Gy IR. Cells were harvested and stained with propidium iodide to determine the distribution of cells across the cell cycle using flow cytometry. The histogram shows the results from B as a (%). **p < 0.01. The results are representative of three independent experiments.

To explore how ITGA6 might contribute to the radioresistance response of breast cancer cells, a protocol was established that allowed cell cycle progression to be monitored by flow cytometry following ITGA6 depletion using shRNA and IR exposure in MCF7, SKBR-3 and MDA-MB-231 cells. Using a dose range of IR, it was determined that all cell lines exhibited a major increase in the G2/M fraction 24 hours after exposure with 8Gy IR. Hence, in these experiments, cells were first transfected with control (shCtrl) or ITGA6shRNA and then, after 48 hours, they were either untreated or exposed to 8 Gy IR. Following a further 24 hours, they were collected for analysis by propidium iodide (PI)-based flow cytometry. Using this protocol, no significant change was observed in the fraction of cycling cells in the G2/M phase of the cell cycle after ITGA6 depletion without IR. However, following IR exposure, cells depleted of ITGA6 exhibited a substantial increase in the G2/M fraction as compared to cells depleted with control oligonucleotides (Fig. 4).

### ITGA6 enhanced DNA damage repair in irradiated cells

**Figure 5:**
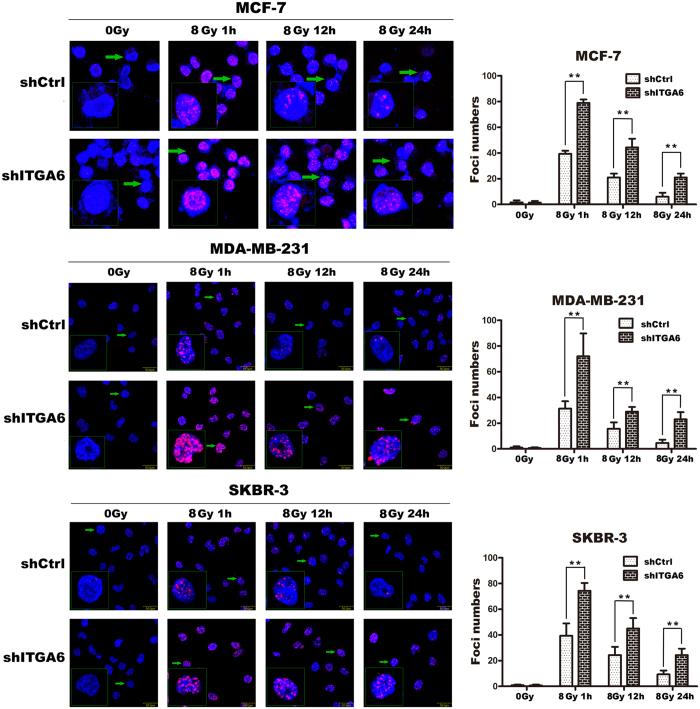
ITGA6 depletion impaired repair of radiation-induced DNA DSBs. (**A**–**C**) Cells were irradiated with a single dose of 8 Gy X-rays followed by collection for immunofluorescence after 1 h, 12 h and 24 h. Nuclear staining was performed using DAPI (blue), and labeling forγ-H2AX is shown as red points (foci). The quantitative analysis of the number of γ-H2AX foci per cell showed that there were an average of over 50 cells per data point. **p < 0.01. The results are representative of three independent experiments.

Altering the frequency of DNA repair activities could also lead to radioresistance. To examine the DSB repair efficiency of ITGA6 knockdown cells compared to control cell lines, phosphorylation of H2AX (γ-H2AX) was assayed. In this assay, the persistence of γ-H2AX foci following IR reflects an impaired cellular capacity to repair DNA DSBs. Thus, γ-H2AX foci were assayed at different time points after the delivery of 8 Gy of IR. As shown in Fig. 4, the γ-H2AX foci disappeared faster in the control cells at the 12 h and 24 h timepoints post-IR compared with the cells expressing shITGA6 that were irradiated. These results suggest that ITGA6 may play a role in enhancing the capacity of cells to repair radiation-induced DNA DSBs (Fig. 5).

### ITGA6 enhanced radioresistance via the PI3K/Akt and MEK/Erk signaling pathways

**Figure 6:**
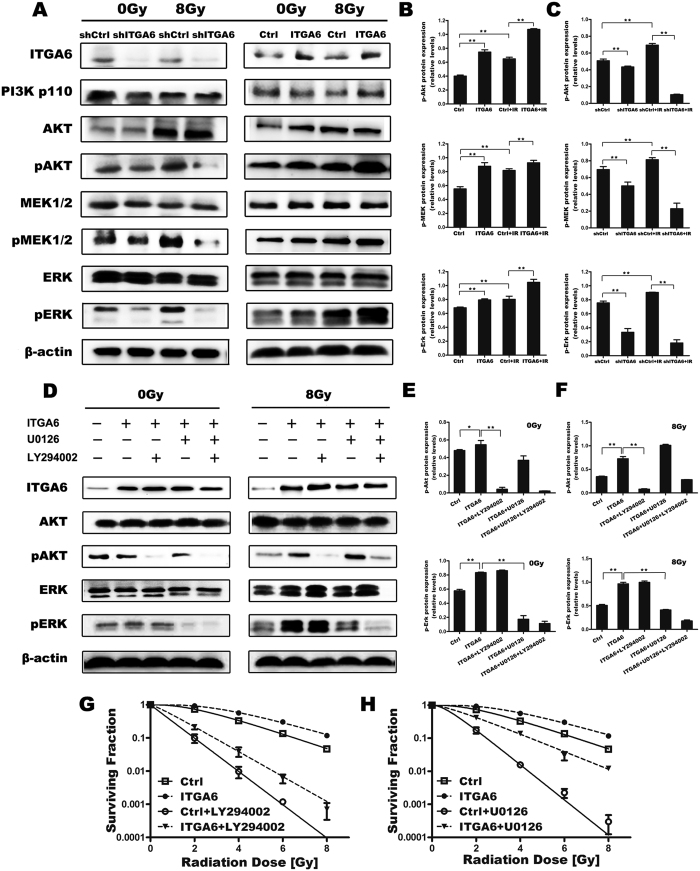
The effect of IR and ITGA6 expression on Akt, MEK and Erk phosphorylation was different between cell lines. (**A**) Cell lysates were collected before and after radiation. The lysates were then immunoblotted using anti-pErk, anti-Erk, anti-pAkt, anti-Akt, anti-pMEK and anti-MEK antibodies. β-actin was used as the loading control. (**B**,**C**) Graphical representation of the relative levels of pAkt, pMEK and pErk. The intensity was quantified using densitometry (software: Image J, NIH). The quantitative data are presented as the means ± SD from three independent experiments (**p < 0.01). (**D**) The cells were treated with 20 μM LY294002 or 10 μM U0126 for 1 h prior to IR and then irradiated with 8 Gy. Total cell lysates were harvested at 1 h after IR, and the lysates were subjected to Western blot analysis using the indicated antibody. (**E,F**) Graphical representation of the relative levels of pAkt and pErk. The intensity was quantified using densitometry (software: Image J, NIH). The quantitative data are presented as the means ± SD from three independent experiments (**p < 0.01). (**G,H**) Cells were treated with control, 20 μM LY294002 and 10 μM U0126 for 1 hrand then irradiated using the indicated dosage. At 4 h after IR, the cells were fed drug-free medium and incubated for another 20 h at 37 °C, after which they were trypsinized and seeded for clonogenic survival assays. Colony-forming efficiency was determined 14d later. The results were pooled from three different experiments.

To further investigate the mechanism underlying ITGA6-induced radiation resistance, we detected the expression of two key genes, Akt and Erk, which are known to be involved in breast tumorigenesis and radioresistance. We first examined the effect of IR on Akt and Erk phosphorylation before and after radiotherapy. As shown in Fig. 6A–C, IR increased the phosphorylation of Akt and Erk in MCF-7 cells. We then investigated whether ITGA6 could modulate phospho-Akt and -Erk expression. Silencing ITGA6 inhibited Akt and Erk phosphorylation when applied before radiotherapy, and this inhibitory effect was not reversed by IR. Conversely, we observed an additive effect whereby radiotherapy increased the expression of p-Akt and p-Erk in ITGA6-overexpressing cells.

We then sought to determine whether ITGA6 modulates radiosensitivity in breast cancer cells via a mechanism involving Akt and Erk signaling. First, the PI3K inhibitor LY294002 was used to inhibit the IR-induced activation of Akt because PI3K is upstream of Akt signaling. MCF-7 Cells were pretreated for1 hr with LY294002 and then irradiated at 0–8 Gy, and their reproductive growth capability was measured using a clonogenic survival assay. As shown in Fig. 5D,E, treatment with LY294002 abolished IR-induced Akt phosphorylation, indicating that this process is dependent upon PI3K. These results were consistent with those described in other reports. In addition, compared to ionizing radiation alone, treatment with LY294002 resulted in significantly higher levels of radiosensitivity in breast cancer cells, and the radioresistance induced by ITGA6 overexpression was restored by inhibiting PI3K/Akt (Fig. 5G). These results indicated that inhibiting PI3K signaling plays an important role in repressing the response of breast cancer cells to IR treatment.

Similarly, we found that the MEK-specific inhibitor U0126 exerted a sensitization effect following irradiation because MEK is upstream of Erk signaling (Fig. 5D,F). Western blot analysis showed that exposing the cells to U0126, either before or after radiation, inhibited the basal level of Erk phosphorylation. As shown in Fig. 5H, inhibiting the MEK/Erk axis had the expected effect of enhancing the radiosensitivity of breast cancer cells. These results indicate that the MEK/Erk mediates the mechanism by which ITGA6 protects against cell damage caused by IR.

## Discussion

Early detection methods and novel therapies have significantly improved the ability to diagnose and treat breast cancer in recent years^2^. However, breast cancer remains a major health threat because of its high incidence, and it continues to be the second leading cause of cancer-related deaths in women^1^. In this era of personalized medicine, developing molecular markers that can be used to predict the clinical benefits of specific interventions is of critical importance. Specifically, there is an unmet need for predictive biomarkers that can be used to identify which patients are likely to benefit from radiotherapy. Predictive biomarkers are defined as markers that can be used to identify subpopulations of patients who are most likely to respond to a given therapy. Here, we show that ITGA6 is associated with radiation sensitivity and that perturbing this gene is sufficient to alter responses to radiation. We also developed a molecular signature for responsiveness to radiation in breast cancer in which biological characteristics implicated in responsiveness to radiotherapy, including alterations in apoptosis, DNA damage repair and cell-cycle regulation, were enriched. Furthermore, we hypothesized that ITGA6 is able to identify patients that are unlikely to develop local recurrence after radiotherapy and those patients who have a high likelihood of recurrence despite undergoing standard radiotherapy.

Previous studies have shown that ITGA6 is overexpressed in breast carcinoma^6^. Overexpression of the ITGA6 gene promotes cancer cell proliferation^10^,^11^, and a relationship exists between the expression of ITGA6 and radioresistance in prostate cancer and esophageal cancer^14^. Moreover, abnormal ITGA6 expression was shown to be responsible for tumor-like properties in a breast cancer stem cell-like subpopulation^7^,^8^,^9^. These data suggest that ITGA6 serve as “functional” markers for mammary cancer stem cells (MaCSCs) because they play a role in regulating MaCSCs rather than acting simply as surface markers. Our results provide further evidence supporting the notion that blocking ITGA6 expression or using specific agents that target the integrin signaling pathway maximize the efficacy of treatments by sensitizing tumor cells to IR. These data also suggest a potential role for the effect of ITGA6 signaling on MaCSCs in breast cancer resistance to radiotherapy.

Early studies in radiation biology used both *in vitro* and *in vivo* models to show that various determinant factors contribute to differential levels of radiosensitivity. These included both extrinsic and intrinsic factors in addition to the tumor microenvironment (e.g., hypoxia and interactions with stromal elements) and radiation response elements (e.g., radiation-induced apoptosis, cell cycle distribution, antioxidant levels, and DNA repair capacity)^20^,^21^,^22^. The cell cycle phase determines a cell’s relative radiosensitivity, with cells being most radiosensitive in the G2-M phase, less sensitive in the G1 phase, and least sensitive during the latter part of the S phase. This understanding has, therefore, led to the realization that one way in which chemotherapy and fractionated radiotherapy may work better is by partial synchronization of cells in the most radiosensitive phase of the cell cycle. Recent studies have shown that some anticancer-drugs could induce G2/M arrest accompanying the down-regulation of Akt^23^,^24^,^25^. Inhibition of the PI3-Kinase pathway by LY294002 induces a G2 arrest and apoptosis^26^. In our study, following IR exposure, cells depleted of ITGA6 exhibited a substantial increase in the G2/M fraction as compared to cells depleted with control oligonucleotides. ITGA6 depletion inhibited Akt phosphorylation after radiotherapy. Meanwhile, we also found that abrogating radiation-induced pAkt increase radiosensitivity of cancer cells. These results indicated that ITGA6 depletion induced G2/M arrest and apoptosis possibly by down-regulating Akt signaling in human breast cancer cells.

DSBs are the most important cytotoxic lesion induced by IR. Cells are equipped with sensors that detect DNA damage and relay the signal via kinases to executors, who on their turn evoke a process that inhibits cell cycle progression and provokes DNA repair or, if this fails, activate the receptor and/or mitochondrial apoptotic cascade^27^. Preclinical *in vitro* and *in vivo* studies have shown that activated Akt accelerates repair of IR-induced DNA double-strand breaks (DNADSB) and, consequently, improves post-irradiation cell survival^28^. DNA damage most often activates the extrinsic death receptor apoptosis pathway (Fas, CD95, Apo-1) and/or the intrinsic mitochondrial apoptosis pathway. This paradigm receives support from the dual function of Akt^27^. When DNA damage is not repaired, the suppression of apoptosis by Akt is abolished. Our data demonstrate that phosphorylated Akt is sufficient to alter responses to the up- or down regulation of ITGA6 after IR. PI3K inhibitor LY294002 partially antagonized the protective effect of ITGA6 against cell death. This study provides evidence of potential mechanism of ITGA6 with DNA double strand damage repair via Akt pathway in breast cancer cells. In addition, cancer stem cells have emerged as contributors to radioresistance because they preferentially activate the DNA damage checkpoint response and increase DNA repair capacity^29^. ITGA6 serving as “functional” markers for mammary cancer stem cells also may be one of potential mechanism of ITGA6 contributes to DNA double strand damage repair.

The name integrin refers to the function of family members to integrate cell exteriors (e.g., ECM) to the cell interiors (e.g., the cytoskeleton)^15^. Integrins form heterodimeric transmembrane cell-matrix adhesion receptors. Integrins do more than simply attach a cell to its surroundings; they also activate intracellular signaling pathways. FAK activation, which leads to the activation of PI3K, has been extensively documented to be one of the central events that is caused by integrin signaling. Upon its activation via an integrin-mediated cell adhesion mechanism, FAK becomes associated with several SH2 domain-containing molecules, including Src^30^,^31^ and the p85^32^,^33^ subunit of PI3K, at its autophosphorylated Y397 residue. FAK binding to the SH2 domain of Src prevents Src Y527 from binding to it, which relieves an auto-inhibitory interaction and leads to the activation of Src. Conversely, activated Src phosphorylates additional sites on FAK, including the residues Y576 and Y577 in FAK’s kinase activation loop, which leads to a further increase in the activity of FAK. Y925 promotes the binding of the adaptor molecule Grb2, which mediates the activation of Ras-MAPK signaling^34^. The association with FAK and the subsequent activation of PI3K at the autophosphorylated residue Y397 leads to an increase in the production of 3′-phosphorylated phospholipids^35^, which activates Akt kinase. The activation of Akt kinase inhibits apoptosis by regulating various cell death machinery proteins^36^,^37^. PI3K inhibitors have long been known to sensitize cells to or to work in combination with IR to enhance apoptosis in breast cancer cells^16^. Akt has also emerged as a central mediator of resistance. Moreover, extensive evidence indicates that the activation of MAPK/Erk pathways is correlated with tumor progression, resistance to treatment and worse survival inpatients^18^,^19^. Our data demonstrate that both phosphorylated Akt and Erk are sufficient to alter responses to the up- or downregulation of ITGA6 after IR. To further explore the correlation between the expression of ITGA6 and the activation of the PI3K/Akt and MAPK/Erk signaling pathways, we suppressed the function of PI3K/Akt using the PI3K-specific inhibitor LY294002 and the Erk-specific inhibitor U0126. We found that both U0126 and LY294002 partially antagonized the protective effect of ITGA6 against cell death, thereby enhancing the radiosensitivity of the cells.

In summary, this study provides evidence of a mechanism that ITGA6 contributes to radioresistance via an ITGA6/Akt/Erk pathway in breast cancer cells. Larger sample clinical studies are required to verify the clinical significance of ITGA6 as an independent prognostic factors for breast cancer, and an animal experiment requires further study. Gene therapeutic approaches may enhance the benefit of radiotherapy by targeting ITGA6 expression. Alternatively, examining the expression of ITGA6 may provide clinicians with relevant information that may indicate which patients are likely to respond poorly to standard radiotherapy.

## Methods

### Cell Culture

The normal mammary epithelial cell line HBL-100 and a series of breast cancer cell lines, including MCF–7, MDA-MB-231, HCC1937, SKBR-3, and BT549, were obtained from the Cell Bank of the Chinese Academy of Sciences (Shanghai, China). Cells were cultured in RPMI 1640 medium (HyClone) containing 10% fetal bovine serum (FBS) (Gibco). Platinum-A Retroviral Packaging cells (Cell BioLabs) were grown in DMEM medium (HyClone) with 10% FBS (Gibco), 10 μg/μlpuromycin, and 100 μg/μlBlasticidin. Cells were culturesand experiments were performedin a humidified 37 °C incubator with 5% CO_2_.

### Plasmids, shRNA and Transfections

Retroviral vectors were obtained by ligating a human integrin alpha6 cDNA (generous gifted by Dr. A. Sonnenberg, Cancer Institute, Amsterdam, The Netherlands) into the LZRS-IRES-ZEO retroviral vector. The defective retroviral particles that were used to infect human breast cell lines were obtained from the conditioned media of Platinum-A Retroviral Packaging cells (Cell BioLabs) after transfection with LZRS-IRES-zeo or LZRS-IRES-zeo-α6 plasmids. The shRNA targeting the integrin alpha 6 gene and the control shRNA (non-targeting) were synthesized by Genema (Shanghai, China). Using a transfection strategy, we created stable breast cancer cell lines in which ITGA6 was either overexpressed or silenced.

### RNA Extraction, Reverse Transcription (RT) and Real-time PCR

Total RNA was isolated using 1 mL Trizol (Invitrogen) according to the manufacturer’s recommendations. RT-PCR was performed using an ABI Prism7000RT system with SYBR-Green Master Mix (TaKaRa). The following primers were used: integrin α6, GAGCTTTTGTGATGGGCGATT, CTCTCCACCAACTTCATAAGGC; and β-actin, GGACTTCGAGCAAGAGATGG, AGCACTGTGTTGGCGTACAG.

### Western Blot Analysis

Protein extraction from frozen tissues and cell lines was performed using RIPA buffer (Sigma, USA) (RIPA:PMSF = 100:1). Lysates of the same amounts of protein were separated on 8–12% SDS-polyacrylamide gels and then transferred onto nitrocellulose membranes. The following antibodies were purchased: integrin α6 (Abcam, ab124924), ITGA6 G0H3(BD), PI3K p110(Abgent, AP8016c), AKT1 (Abgent, AP7141h), phospho-ATK1(Abgent, AP3434a), MEK1/2(Signalway Antibody), Phospho-MEK1/2(Signalway Antibody), ERK1/2(Abgent, AM2189b), phospho-ERK1/2(Abgent, AP3607a), NF-κB (CST), phospho-NF-κB (CST), caspase 3(CST), γ-H2AX (Abcam), and β-actin (Santa Cruz). Membranes were developed using an ECL detection system after the membranes were incubated with peroxidase-conjugated secondary antibodies.

### Clonogenic Assay

The sensitivity to irradiation of cells in different treatment groups was determined using clonogenic assays after the cells were exposed to variable doses of radiation (0 Gy, 2 Gy, 4 Gy, 6 Gy or 8 Gy) using a linear accelerator. After the cells were incubated for 10–14 days, the colonies that had formed were fixed in methanol and stained with 1%crystal violet. Only the colonies that consisted of more than 80 cells were counted. The data were fitted to a linear-quadratic model using Sigma plot software. Survival curves were generated, and radiosensitivity parameters were calculated. The experiments were repeated three times.

### Cell Apoptosis Analysis

The effect on cell apoptosis of integrin a6 knockdown or overexpression was evaluated using flow cytometry. Briefly, cell apoptosis was analyzed using an Annexin V-APC/7-AAD apoptosis kit (KeyGen Biotech) according to the manufacturer’s instructions.

### Cell Cycle Analysis

Cell cycle analyses were performed using Cell Cycle and Apoptosis Analysis Kits (Beyotime, China) according to the manufacturer’s instructions. A FACScan cytometer (BD, USA) was used to analyze cells and to determine the percentage of cells that were in the G0/G1, S, and G2/M phases of the cell cycle using ModFit Software.

### Immunofluorescence microscopy for γH2AX

A total of 2 × 10^4 ^cells were seeded into 35 mm dishes in which a glass coverslip was contained in each well. After the cells were exposed to 0 or 8 Gy irradiation, the slides were air-dried and fixed for 15–20 min in 4% paraformaldehyde in TBS. The cells were then rinsed in TBS, placed in 0.2% TritonX-100 in 4 °C methanol for 10 min, rinsed, and placed in TBS plus 5% bovine serum albumin for 1 h. Finally, the cells were incubated overnight with diluted anti-phospho-histone H2AX mAb (Abcam, diluted 1:500). The slides were washed and incubated with cy3-conjugated anti-mouse goat F(ab′) fragments (Santa Cruz) diluted to 1:100 for 1 h at room temperature. The slides were then rinsed and immersed in Hoechst reagent for 15 min. To prevent bias in selecting cells that displayed foci, over 800 randomly selected cells were counted. Cells with three or more foci of any size were classified as positive. The experiments were repeated at least three times.

### Statistics

The data analysis was performed using a 2-tailed Student’s t-test with pooled variance. The data are expressed as the mean ± SD of at least three sampled replicates unless stated otherwise. In the Figures, *denotes P < 0.05, **denotes P < 0.01, and ^#^denotes P > 0.05.

## Additional Information

**How to cite this article**: Hu, T. *et al*. Integrin α6/Akt/Erk signaling is essential for human breast cancer resistance to radiotherapy. *Sci. Rep.* **6**, 33376; doi: 10.1038/srep33376 (2016).

## References

[b1] Siegel R. L., Miller K. D. & Jemal A. Cancer statistics, 2015. CA: a cancer journal for clinicians 65, 5–29 (2015). 25559415 10.3322/caac.21254

[b2] Group E. B. C. T. C. Effects of radiotherapy and of differences in the extent of surgery for early breast cancer on local recurrence and 15-year survival: an overview of the randomised trials. The Lancet 366, 2087–2106 (2006). 10.1016/S0140-6736(05)67887-7 16360786

[b3] Park C. C. . Outcome at 8 years after breast-conserving surgery and radiation therapy for invasive breast cancer: influence of margin status and systemic therapy on local recurrence. Journal of Clinical Oncology 18, 1668–1675 (2000). 10764427 10.1200/JCO.2000.18.8.1668

[b4] Hehlgans S., Haase M. & Cordes N. Signalling via integrins: implications for cell survival and anticancer strategies. Biochimica et Biophysica Acta (BBA)-Reviews on Cancer 1775, 163–180 (2007). 17084981 10.1016/j.bbcan.2006.09.001

[b5] Giancotti F. G. & Ruoslahti E. Integrin signaling. Science 285, 1028–1033 (1999). 10446041 10.1126/science.285.5430.1028

[b6] Friedrichs K. . High expression level of α6 integrin in human breast carcinoma is correlated with reduced survival. Cancer Research 55, 901–906 (1995). 7850807

[b7] Cariati M. . Alpha‐6 integrin is necessary for the tumourigenicity of a stem cell‐like subpopulation within the MCF7 breast cancer cell line. International Journal of Cancer 122, 298–304 (2008). 17935134 10.1002/ijc.23103

[b8] Mostert B. . CD49f-based selection of circulating tumor cells (CTCs) improves detection across breast cancer subtypes. Cancer letters 319, 49–55 (2012). 22202642 10.1016/j.canlet.2011.12.031

[b9] Lo P.-K. . CD49f and CD61 identify Her2/neu-induced mammary tumor-initiating cells that are potentially derived from luminal progenitors and maintained by the integrin–TGFβ signaling. Oncogene 31, 2614–2626 (2012). 21996747 10.1038/onc.2011.439 PMC3260386

[b10] Shaw L. M., Chao C., Wewer U. M. & Mercurio A. M. Function of the integrin α6β1 in metastatic breast carcinoma cells assessed by expression of a dominant-negative receptor. Cancer research 56, 959–963 (1996). 8640785

[b11] Wewer U. M., Shaw L. M., Albrechtsen R. & Mercurio A. M. The integrin alpha 6 beta 1 promotes the survival of metastatic human breast carcinoma cells in mice. The American journal of pathology 151, 1191 (1997). 9358743 PMC1858063

[b12] Mukhopadhyay R., Theriault R. L. & Price J. E. Increased levels of α6 integrins are associated with the metastatic phenotype of human breast cancer cells. Clinical & experimental metastasis 17, 323–330 (1999). 10.1023/a:1006659230585 10545019

[b13] Im Kim H., Huang H., Cheepala S., Huang S. & Chung J. Curcumin inhibition of integrin (α6β4)-dependent breast cancer cell motility and invasion. Cancer Prevention Research 1, 385–391 (2008). 19138983 10.1158/1940-6207.CAPR-08-0087

[b14] Pawar S. C. . Alpha 6 integrin cleavage: sensitizing human prostate cancer to ionizing radiation. International journal of radiation biology 83, 761–767 (2007). 18058365 10.1080/09553000701633135 PMC2732343

[b15] Lambert A. W., Ozturk S. & Thiagalingam S. Integrin signaling in mammary epithelial cells and breast cancer. ISRN oncology 2012 (2012). 10.5402/2012/493283 PMC3317013 22523705

[b16] Liang K. . Targeting the phosphatidylinositol 3-kinase/Akt pathway for enhancing breast cancer cells to radiotherapy1. Molecular cancer therapeutics 2, 353–360 (2003). 12700279

[b17] Albert J. M., Kim K. W., Cao C. & Lu B. Targeting the Akt/mammalian target of rapamycin pathway for radiosensitization of breast cancer. Molecular Cancer Therapeutics 5, 1183–1189 (2006). 16731750 10.1158/1535-7163.MCT-05-0400

[b18] McCubrey J. A. . Roles of the Raf/MEK/ERK pathway in cell growth, malignant transformation and drug resistance. Biochimica et Biophysica Acta (BBA)-Molecular Cell Research 1773, 1263–1284 (2007). 17126425 10.1016/j.bbamcr.2006.10.001 PMC2696318

[b19] De Luca A., Maiello M. R., D’Alessio A., Pergameno M. & Normanno N. The RAS/RAF/MEK/ERK and the PI3K/AKT signalling pathways: role in cancer pathogenesis and implications for therapeutic approaches. Expert opinion on therapeutic targets 16, S17–S27 (2012). 22443084 10.1517/14728222.2011.639361

[b20] Guichard M., Dertinger H. & Malaise E. Radiosensitivity of four human tumor xenografts. Influence of hypoxia and cell-cell contact. Radiation research 95, 602–609 (1983). 6611864

[b21] Bravard A. . Correlation between antioxidant status, tumorigenicity and radiosensitivity in sister rat cell lines. Carcinogenesis 23, 705–711 (2002). 12016141 10.1093/carcin/23.5.705

[b22] Al-Assar O. . Breast cancer stem-like cells show dominant homologous recombination due to a larger S-G2 fraction. Cancer biology & therapy 11, 1028–1035 (2011). 21558789 10.4161/cbt.11.12.15699

[b23] Weir N. M. . Curcumin induces G2/M arrest and apoptosis in cisplatin-resistant human ovarian cancer cells by modulating Akt and p38 MAPK. Cancer biology & therapy 6, 178–184 (2007). 17218783 10.4161/cbt.6.2.3577 PMC1852522

[b24] Katayama K., Fujita N. & Tsuruo T. Akt/protein kinase B-dependent phosphorylation and inactivation of WEE1Hu promote cell cycle progression at G2/M transition. Molecular and cellular biology 25, 5725–5737 (2005). 15964826 10.1128/MCB.25.13.5725-5737.2005 PMC1156994

[b25] Xu X., Zhang Y., Qu D., Jiang T. & Li S. Osthole induces G2/M arrest and apoptosis in lung cancer A549 cells by modulating PI3K/Akt pathway. Journal of Experimental & Clinical Cancer Research 30, 1 (2011). 21447176 10.1186/1756-9966-30-33 PMC3073874

[b26] Shtivelman E., Sussman J. & Stokoe D. A role for PI 3-kinase and PKB activity in the G2/M phase of the cell cycle. Current Biology 12, 919–924 (2002). 12062056 10.1016/s0960-9822(02)00843-6

[b27] Roos W. P. & Kaina B. DNA damage-induced cell death: from specific DNA lesions to the DNA damage response and apoptosis. Cancer letters 332, 237–248 (2013). 22261329 10.1016/j.canlet.2012.01.007

[b28] Toulany M. & Rodemann H. P. Potential of Akt mediated DNA repair in radioresistance of solid tumors overexpressing erbB-PI3K-Akt pathway. Translational Cancer Research 2, 190–202 (2013).

[b29] Brunner T. B., Kunz-Schughart L. A., Grosse-Gehling P. & Baumann M. In Seminars in radiation oncology. 151–174 (Elsevier). 10.1016/j.semradonc.2011.12.003 22385922

[b30] Schaller M. . Autophosphorylation of the focal adhesion kinase, pp125FAK, directs SH2-dependent binding of pp60src. Molecular and cellular biology 14, 1680–1688 (1994). 7509446 10.1128/mcb.14.3.1680-1688.1994 PMC358526

[b31] Xing Z. . Direct interaction of v-Src with the focal adhesion kinase mediated by the Src SH2 domain. Molecular biology of the cell 5, 413–421 (1994). 8054685 10.1091/mbc.5.4.413 PMC301051

[b32] Chen H.-C. & Guan J.-L. Association of focal adhesion kinase with its potential substrate phosphatidylinositol 3-kinase. Proceedings of the National Academy of Sciences 91, 10148–10152 (1994). 10.1073/pnas.91.21.10148 PMC44975 7937853

[b33] Chen H.-C., Appeddu P. A., Isoda H. & Guan J.-L. Phosphorylation of tyrosine 397 in focal adhesion kinase is required for binding phosphatidylinositol 3-kinase. Journal of Biological Chemistry 271, 26329–26334 (1996). 8824286 10.1074/jbc.271.42.26329

[b34] Schlaepfer D. D., Hanks S. K., Hunter T. & van der Geer P. Integrin-mediated signal transduction linked to Ras pathway by GRB2 binding to focal adhesion kinase (1994). 10.1038/372786a0 7997267

[b35] Reiske H. R. . Requirement of phosphatidylinositol 3-kinase in focal adhesion kinase-promoted cell migration. Journal of Biological Chemistry 274, 12361–12366 (1999). 10212207 10.1074/jbc.274.18.12361

[b36] Hennessy B. T., Smith D. L., Ram P. T., Lu Y. & Mills G. B. Exploiting the PI3K/AKT pathway for cancer drug discovery. Nature reviews Drug discovery 4, 988–1004 (2005). 16341064 10.1038/nrd1902

[b37] Luo J., Manning B. D. & Cantley L. C. Targeting the PI3K-Akt pathway in human cancer: rationale and promise. Cancer cell 4, 257–262 (2003). 14585353 10.1016/s1535-6108(03)00248-4

